# An Exploratory Study of Factors Associated with Medical Students’ Self-Reported Online Innovative Behavior in Online Learning Environments: A Mediation Analysis

**DOI:** 10.3390/bs16081376

**Published:** 2026-08-11

**Authors:** Yao Xiao, Qing Hou, Shudi Li, Shuyuan Sun, Qiaoling Miao, Yueyi Zhang, Bowen Liu

**Affiliations:** 1 Faculty of Artificial Intelligence in Education, Central China Normal University, Luoyu Road No. 152, Wuhan 430079, China; profilerling@mails.ccnu.edu.cn (Y.X.); 2011050@wtu.edu.cn (Q.H.); lsd@mails.ccnu.edu.cn (S.L.); mql@mails.ccnu.edu.com (Q.M.); 2 School of Computer Science, Central China Normal University, Nanhu Complex Building, Office 7065, Wuhan 430079, China; ssy199191@ccnu.edu.cn; 3 The Second School of Clinical Medicine, Southern Medical University, Gongye Avenue, No. 253, Guangzhou 510280, China; irene_zhang0717@163.com

**Keywords:** self-reported online innovative behavior, digital resilience, technological self-efficacy, online learning engagement, self-directed learning behaviors, mediation effect, medical students

## Abstract

**Background:** Cultivating medical students’ innovative behavior is crucial for talent development in medical education. However, the factors associated with medical students’ innovative behavior are still unclear. The aim was to examine factors associated with self-reported online innovative behavior of medical students. **Methods:** A cross-sectional electronic survey was conducted at a public medical university in China. A total of 371 medical undergraduates participated in the study. Data were collected with questionnaires on students’ abilities scales and analyzed with SPSS 26 and AMOS 26. The associations among constructs were analyzed through structural equation modeling. **Results:** Results showed that both digital resilience and technological self-efficacy were positively associated with medical students’ self-reported online innovative behavior. Statistically significant indirect associations were found between digital resilience and self-reported online innovative behavior through online learning engagement and self-directed learning behaviors. Statistically significant indirect associations were also found between technological self-efficacy and self-reported online innovative behavior through online learning engagement and self-directed learning behaviors. **Conclusions:** Digital resilience and technological self-efficacy were directly and indirectly associated with medical students’ self-reported online innovative behavior in online learning environments. The results provide useful empirical insights for understanding students’ self-reported online innovative behavior in online learning environments.

## 1. Introduction

The ultimate goal of medical education is to cultivate medical talents. In recent years, online learning has been widely used in medical education, and has significantly improved the quality of medical talent training (Zhao et al., 2018). Compared with traditional teaching, online learning supported by digital technology can facilitate more flexible adjustment of teaching modes and implementation of teaching strategies (D. Jin & Li, 2020). It can also embed functions that are conducive to observing and improving students’ learning effects in real time, such as learning behaviors analysis, learning content analysis, and student interaction analysis (Kui et al., 2022). At present, online learning is generally considered to be associated with improvements in students’ learning efficiency and quality (Ahmmed et al., 2022).

With the continuous advancement of medical science and healthcare technology, along with increasing public demand for high-quality healthcare services, the healthcare sector increasingly requires future healthcare professionals to develop comprehensive competencies and high-level capabilities, such as innovation literacy, digital literacy, clinical reasoning, and problem-solving skills (R. Liu et al., 2025). Innovation literacy encompasses innovation-related cognition, motivation, and behavior, whereas innovative behavior represents the process through which individuals translate these internal attributes into concrete actions (Gao et al., 2025). Innovative behavior is defined as a series of actions through which individuals generate and attempt to implement novel and valuable ideas within specific environments (Scott & Bruce, 1994). It is manifested in identifying problems, proposing new perspectives or solutions, and facilitating the realization of these ideas through practical actions (Bao et al., 2024). Consequently, innovative behavior can be viewed as an observable manifestation of students’ innovation literacy in specific learning environments and provide an important behavioral indicator for understanding the development of such literacy. Individuals who engage in innovative behavior not only enhance their own performance in specific fields but also stimulate transformation and progress within those domains (Rattanawichai et al., 2023).

To meet increasingly complex healthcare demands, medical education must not only strengthen students’ professional knowledge and clinical skills but also emphasize the development of their innovation literacy. Fostering innovative behavior can help medical students apply innovative thinking and problem-solving strategies to medical learning, clinical reasoning, and future professional practice (Wu et al., 2025). However, the shift from traditional instructional settings to online learning environments has profoundly changed teaching methods and learning patterns in medical education. Although online learning transcends spatial and temporal constraints and provides abundant digital resources, its limited face-to-face interaction, hands-on practice, and real-time feedback may constrain students’ opportunities to generate innovative ideas and translate them into practical learning actions. Therefore, effectively fostering innovative behavior among medical students in online learning environments has become an important issue in medical education.

Previous studies have shown that students with high digital resilience and self-directed learning ability can have higher learning engagement and can better adapt to online learning environments (F. Li et al., 2025). Learning engagement has also been identified as a key factor associated with innovative behavior (W. Huang & Zhang, 2024). Therefore, digital resilience, self-directed learning behaviors, and learning engagement constitute important factors associated with students’ self-reported online innovative behavior in online learning environments. In addition, technological self-efficacy is also a crucial factor for students in online learning environments. Related studies have shown that students with high technological self-efficacy can better use online resources, focus more on current learning and make wise decisions in online learning environments (Mubarak & Selimin, 2023). Therefore, technological self-efficacy can be regarded as an important factor associated with students’ creativity and innovation in online learning environments (Mubarak & Selimin, 2024).

Previous studies have suggested that students with greater resilience and self-efficacy tend to demonstrate stronger innovative thinking and more proactive innovative behavior during the learning process. However, the associations of digital resilience and technological self-efficacy with students’ online innovative behavior in online learning environments, as well as the potential mediating roles of online learning engagement and self-directed learning behaviors, have not been fully explored. The research examined how digital resilience and technological self-efficacy were associated with medical students’ self-reported online innovative behavior in online learning environments. On the basis, we further examined whether online learning engagement and self-directed learning behaviors mediated these associations. These findings provide empirical evidence for understanding the factors associated with medical students’ self-reported online innovative behavior in online learning environments.

## 2. Literature Review and Research Hypotheses

**Figure 1:**
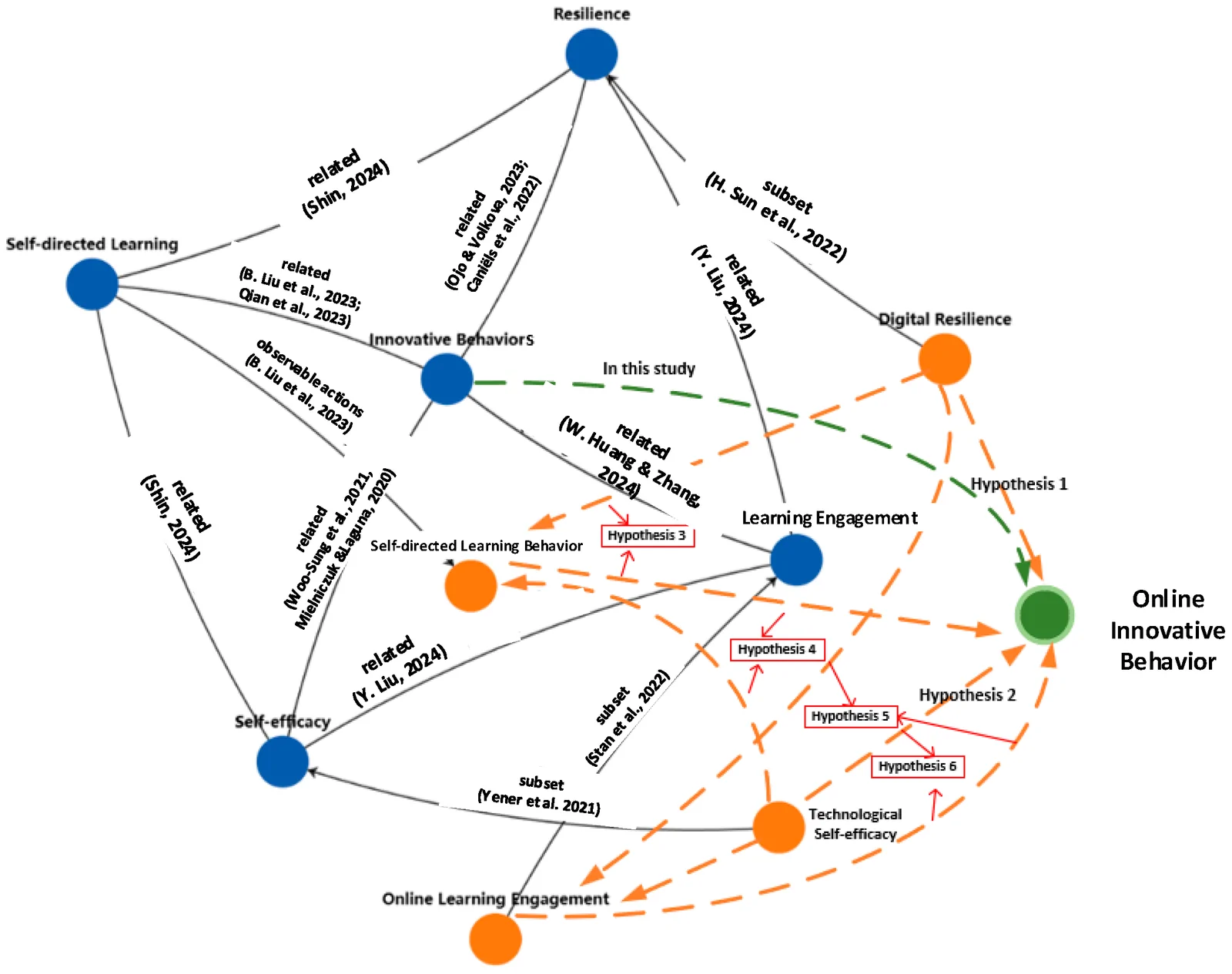
Nomological network map of related literature in online innovative behavior (Shin, 2024; B. Liu et al., 2023; Qian et al., 2023; Ojo & Volkova, 2023; Caniëls et al., 2022; Woo-Sung et al., 2021; Mielniczuk & Laguna, 2020; W. Huang & Zhang, 2024; Y. Liu, 2024; Yener et al., 2021; Stan et al., 2022; H. Sun et al., 2022).

To identify factors associated with students’ online innovative behavior in online learning environments and develop the research hypotheses, a literature review centered on online innovative behavior was conducted. After retrieving and reviewing the relevant literature on the constructs and their associations, a nomological network was constructed (Cronbach & Meehl, 1955; Öztüre Yavuz et al., 2024), as shown in Figure 1.

The nodes in Figure 1 are the constructs covered in the research, and the edges represent the relationships among the constructs and the literature supporting these relationships. As shown in Figure 1, there is a correlation between student resilience, self-efficacy, self-directed learning, learning engagement, and innovative behavior. However, in online learning environments, relatively few studies have examined the relationships of students’ digital resilience, technological self-efficacy, online learning engagement, and self-directed learning behaviors with online innovative behavior. The literature review and nomological network provided the basis for developing the research hypotheses and hypothesized model.

### 2.1. The Relationship Between Digital Resilience and Online Innovative Behavior

Resilience refers to individuals’ ability to adapt positively when facing stress, adversity, or challenging learning environments (Fleming & Ledogar, 2008; Charoensap-Kelly et al., 2021; Ojo & Volkova, 2023). Previous studies have suggested that resilience is related to innovation (Sweetman et al., 2011), especially when people face challenging learning environments (Caniëls et al., 2022). Digital resilience is a specific form of resilience and plays a key role in whether students can adapt after transitioning from traditional scenarios to online learning environments (H. Sun et al., 2022). Based on previous studies, students’ digital resilience is expected to be directly associated with their self-reported online innovative behavior in online learning environments. Therefore, the following hypothesis was proposed:

**Hypothesis** **1.** *Students’ digital resilience is positively associated with their self-reported online innovative behavior in online learning environments.*

### 2.2. The Relationship Between Technological Self-Efficacy and Online Innovative Behavior

Self-efficacy is a person’s belief that he or she can cope with possible difficulties when completing specific tasks or specific behaviors (Bandura, 1977). According to social cognitive theory, people with high self-efficacy believe that they can complete tasks through personal abilities. Even in the face of difficulties, they will work hard to improve their abilities, otherwise they will choose to escape (Woo-Sung et al., 2021). Innovative behavior is often accompanied by difficulties and risks and can be regarded as a challenging activity. Therefore, self-efficacy is expected to be associated with students’ innovative behavior (Mielniczuk & Laguna, 2020; Ng & Lucianetti, 2016). Technological self-efficacy is a specific form of self-efficacy, which means a person’s belief that he or she can use technology to complete corresponding tasks. People with high technological self-efficacy can better use technology to achieve their goals (Yener et al., 2021). In online learning environments, students need to learn to use the relevant technology to operate the learning platform functions to engage in learning and innovative behavior. In addition, existing studies have shown that students with high technological self-efficacy tend to show stronger innovative capabilities (Al-Hattami, 2025). Therefore, the following hypothesis was proposed:

**Hypothesis** **2.** *Students’ technological self-efficacy is positively associated with their self-reported online innovative behavior in online learning environments.*

### 2.3. The Mediating Role of Self-Directed Learning Behaviors

Self-directed learning refers to a process in which learners can independently control their own learning content, learning goals and learning methods to ensure that they can acquire new knowledge and complete tasks (Morris, 2019). Self-directed learning includes both intrinsic motivation and externally observable behaviors (Song & Hill, 2007), and has been found to be positively associated with learners’ creativity and innovative behavior (B. Liu et al., 2023). In research related to the intrinsic motivation of autonomous learning, learners’ resilience and self-efficacy have been found to be significantly associated with self-directed learning ability (Shin, 2024). Therefore, based on the aim of the research to examine the associations of digital resilience and technological self-efficacy with medical students’ self-reported online innovative behavior in online learning environments, the following hypothesis was proposed:

**Hypothesis** **3.** *Students’ self-directed learning behaviors mediate the association between digital resilience and self-reported online innovative behavior.*

**Hypothesis** **4.** *Students’ self-directed learning behaviors mediate the association between technological self-efficacy and self-reported online innovative behavior.*

### 2.4. The Mediating Role of Online Learning Engagement

Learning engagement is the tendency of people to have a persistent and positive emotional state during learning activities (Schaufeli et al., 2002). Learning engagement is important for learners’ acquisition of professional knowledge and development of professional abilities, which are closely related to innovation-related learning outcomes (W. Huang et al., 2019; W. Huang & Zhang, 2024). Online learning engagement, as a manifestation of learners’ learning engagement in online learning environments (Stan et al., 2022), is expected to be associated with students’ self-reported online innovative behavior. In addition, previous studies focusing on students’ learning engagement have shown that students’ resilience and self-efficacy are positively associated with learning engagement (Y. Liu, 2024). Therefore, the following hypothesis was proposed:

**Hypothesis** **5.** *Students’ online learning engagement mediates the association between digital resilience and self-reported online innovative behavior.*

**Hypothesis** **6.** *Students’ online learning engagement mediates the association between technological self-efficacy and self-reported online innovative behavior.*

### 2.5. The Mediation Model

**Figure 2:**
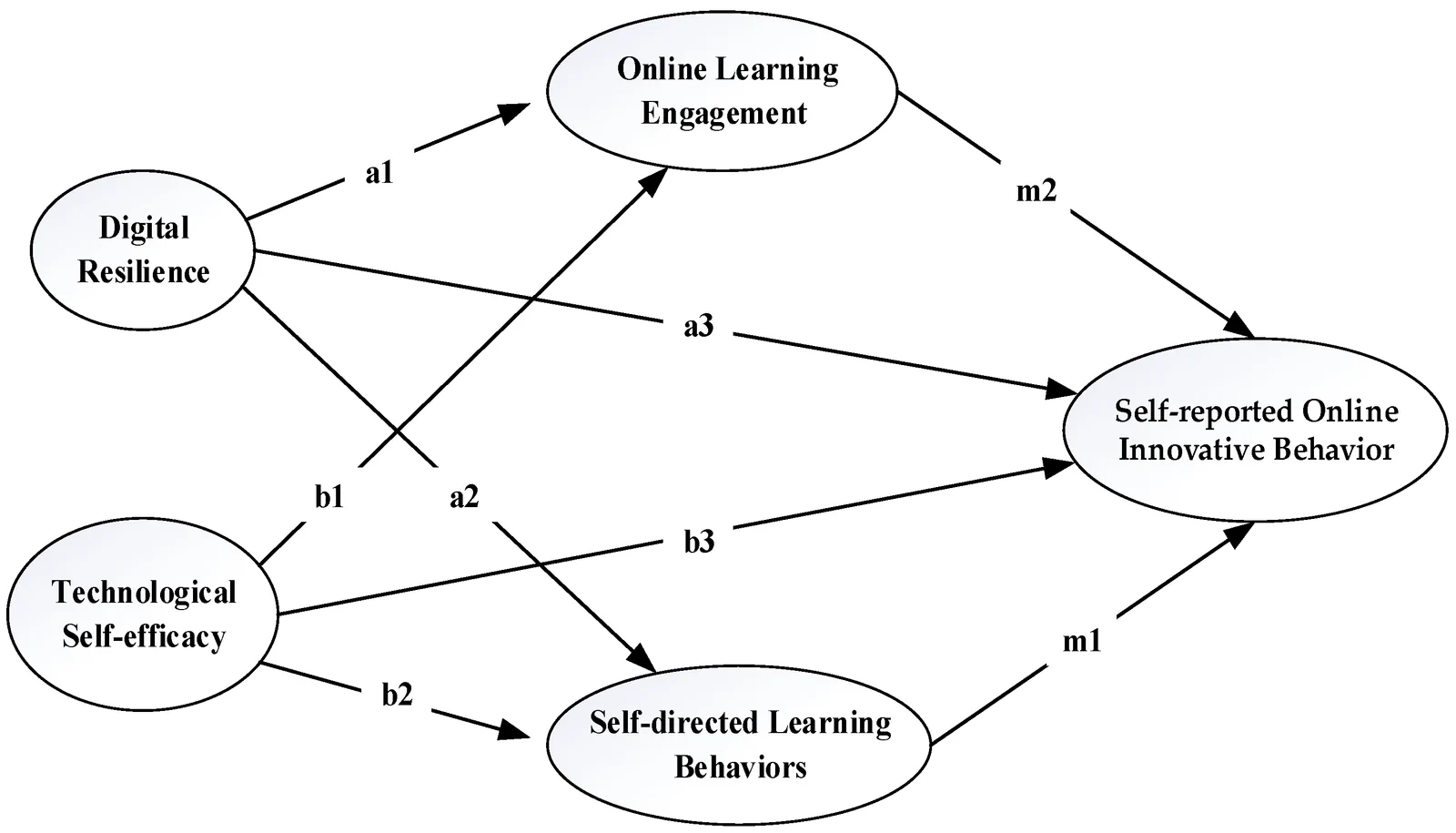
Hypothesis model.

Based on the previous literature review and hypotheses, the mediation model is finally obtained as shown in Figure 2.

Based on the model, the following sections describe the research context, sample information, measurement tools, quantitative research methods based on the model, and the results of testing the relationship among the constructs.

## 3. Materials and Methods

### 3.1. Research Context

The research was conducted at a public medical university in China, which adopted a hybrid teaching model that combines traditional classrooms and online learning to provide students with a more flexible way of learning. Online learning is completed through MOOC, Zhihuishu Online Education Platform, Changjiang RainClassroom, SuperStarLearn, and Tencent Conference. On MOOC and Zhihuishu, students can watch teaching videos and complete corresponding quizzes, and can communicate with teachers and classmates by leaving messages. Changjiang RainClassroom and SuperStarLearn include learning resources corresponding to the course, such as course videos, courseware, and interactive questions. In addition, online learning also includes online live courses, which are completed through Tencent Conference.

### 3.2. Participants and Data Collection

Participants were undergraduate medical students who engaged in online learning during the fall semester of 2024. A convenience sampling method was employed. Recruitment took place from 18 October to 3 November 2024. The questionnaire link was distributed to undergraduate students from different grade levels and majors through class-based online communication channels. A total of 519 students were invited to participate. Participants met the following inclusion criteria: (1) Being currently enrolled as undergraduate students at the institution. (2) Having taken at least one online course during the semester. (3) Voluntarily agreeing to participate in the survey.

Participants completed an anonymous online questionnaire via Wenjuanxing, which is a widely used online survey platform in China. The questionnaire measured digital resilience, technological self-efficacy, online learning engagement, self-directed learning behaviors, and self-reported online innovative behavior.

A total of 410 questionnaires were returned, yielding a response rate of 79.0%. Before data analysis, the questionnaires were checked. In the context of multi-item scales, providing identical responses across all items is commonly referred to as straightlining and is often considered an indication of survey responses of low quality (Aarø et al., 2022). Based on the screening procedure, 39 questionnaires were excluded, leaving 371 valid questionnaires for analysis. The valid questionnaire rate was 90.5% of the returned questionnaires, and the effective response rate among the invited students was 71.5%. Participants ranged in age from 17 to 23 years, with a mean age of 18 years. Among the 371 medical students, 162 were male (43.7%) and 209 were female (56.3%). By year of study, 188 were first-year students (50.7%), 141 were second-year students (38.0%), 30 were third-year students (8.1%), and 12 were fourth-year students (3.2%). All participants completed the questionnaire voluntarily. In order to protect the privacy of participants, each questionnaire was submitted anonymously.

The research received approval from the ethics committee of a public university in China (No. CCNU-IRB-202409002A). The approved protocol involved an anonymous, minimal risk online questionnaire survey among undergraduate medical students. Before completing the questionnaire, all participants were informed of the purpose of the study, the voluntary nature of participation, the anonymity of the survey, the confidentiality of their responses, and their right to withdraw at any time. All participants provided informed consent electronically before proceeding to the questionnaire.

### 3.3. Measures

#### 3.3.1. Digital Resilience Scale

The digital resilience scale in online learning environments was adapted from the Connor–Davidson Resilience Scale (CD-RISC) (Connor & Davidson, 2003) as modified by Kurniadi et al. (2022). The five dimensions of digital resilience reported by Kurniadi et al. (2022) were used as conceptual references for adapting items related to digital resilience for online learning environments. These dimensions were used to organize the adapted items, while digital resilience was modeled as a single latent construct.

The adaptation process was guided by an associate professor with a Ph.D. in educational technology and 1.5 years of visiting experience in English-speaking countries. The item wording was revised to refer explicitly to online learning environments. The complete set of adapted items is presented in Table 1. Each item was scored on a 5-point Likert scale, ranging from 1 (strongly disagree) to 5 (strongly agree). The Cronbach’s alpha of the scale was 0.819, indicating good internal consistency.

#### 3.3.2. Technological Self-Efficacy Scale

Technological self-efficacy was measured using a scale proposed by C.-H. Wang et al. (2013). C.-H. Wang et al. (2013) proposed that technological self-efficacy can be summarized into four dimensions, namely the use of online learning platforms, the content of online learning platforms, the functional interaction of online learning platforms, and the discussion on online learning platforms. These dimensions were used as conceptual references for adapting items related to technological self-efficacy for online learning environments. These dimensions were used to organize the adapted items, while technological self-efficacy was modeled as a single latent construct.

The adaptation process was guided by an associate professor with a Ph.D. in educational technology and 1.5 years of visiting experience in English-speaking countries. The item wording was revised to refer explicitly to online learning environments. The complete set of adapted items is presented in Table 2. Each item was scored using a 5-point Likert scale, ranging from 1 (strongly disagree) to 5 (strongly agree). The Cronbach’s alpha of the scale was 0.871, indicating good internal consistency.

#### 3.3.3. Online Learning Engagement Scale

Online learning engagement was measured using the scale proposed by J. C.-Y. Sun and Rueda (2012). The three dimensions of online learning engagement reported by J. C.-Y. Sun and Rueda (2012), namely behavioral engagement, emotional engagement, and cognitive engagement, were used as conceptual references for adapting items related to online learning engagement for online learning environments. These dimensions were used to organize the adapted items, while online learning engagement was modeled as a single latent construct.

The adaptation process was guided by an associate professor with a Ph.D. in educational technology and 1.5 years of visiting experience in English-speaking countries. The item wording was revised to refer explicitly to online learning environments. The complete set of adapted items is presented in Table 3. Each item was scored using a 5-point Likert scale, ranging from 1 (strongly disagree) to 5 (strongly agree). The Cronbach’s alpha of the scale was 0.816, indicating good internal consistency.

#### 3.3.4. Self-Directed Learning Behaviors Scale

Self-directed learning behaviors was measured using the scale proposed by Lin et al. (2017). The four dimensions of self-directed learning behaviors reported by Lin et al. (2017), namely goal-setting, task strategies, help-seeking, and self-evaluation, were used as conceptual references for adapting items related to self-directed learning behaviors for online learning environments. These dimensions were used to organize the adapted items, while self-directed learning behaviors was modeled as a single latent construct.

The adaptation process was guided by an associate professor with a Ph.D. in educational technology and 1.5 years of visiting experience in English-speaking countries. The item wording was revised to refer explicitly to online learning environments. The complete set of adapted items is presented in Table 4. Each item was scored on a 5-point Likert scale, ranging from 1 (strongly disagree) to 5 (strongly agree). The Cronbach’s alpha of the scale was 0.856, indicating good internal consistency.

#### 3.3.5. Self-Reported Online Innovative Behavior

The scale for medical students’ self-reported online innovative behavior was based on the innovative behavior scale developed by Iddris et al. (2026) and incorporated the adaptation for online learning proposed by J. Wang et al. (2025). The original scale developed by Iddris et al. (2026) was designed to assess students’ innovative behavior in higher education settings, focusing on the innovative actions and attitudes that students demonstrate during learning and task completion.

The research examined medical students’ self-reported online innovative behavior and its associated factors. The adaptation retained the core measurement content of the original scale and contextualized the items within online learning environments to assess medical students’ self-reported online innovative behavior. J. Wang et al. (2025) previously employed the construct of online innovative behavior to examine its mediating role between online learning readiness and online learning engagement. Therefore, adapting students’ innovative behavior to online learning is supported by both theoretical consistency and prior methodological precedent.

The five items of the innovative behavior scale developed by Iddris et al. (2026) were used as source items for adapting items related to self-reported online innovative behavior for online learning environments. The adapted items were used as observed indicators of self-reported online innovative behavior as a single latent construct. The adaptation process was guided by an associate professor with a Ph.D. in educational technology and 1.5 years of visiting experience in English-speaking countries. The item wording was revised to refer explicitly to online learning environments. The complete set of adapted items is presented in Table 5. Each item was scored using a 5-point Likert scale, ranging from 1 (strongly disagree) to 5 (strongly agree). The Cronbach’s alpha of the scale was 0.816, indicating good internal consistency.

The measure assessed medical students’ self-reported online innovative behavior, not objectively observed innovative outcomes, products, or achievements. Therefore, the scale results should be interpreted as students’ subjective reports of their tendencies toward, and performance of, innovative behavior in online learning.

#### 3.3.6. Questionnaire Language, Translation and Scale Adaptation

All participants were Chinese medical students. Therefore, a Chinese-language questionnaire was used for the survey. For scales originally developed in English or other foreign languages, the research team translated the items into Chinese and reviewed them to ensure semantic clarity, conceptual consistency, and contextual appropriateness for Chinese medical students in online learning settings. A translation and back-translation procedure was conducted. The items were first translated into Chinese by one researcher and then back-translated into English by another researcher. The researchers involved in the translation and back-translation were Master’s students specializing in educational technology who had passed the College English Test Band 6. The translation and back-translation processes were conducted independently. The research team compared the back-translated English items with the original items to identify possible discrepancies in meaning, wording, or contextual appropriateness. Any discrepancies were discussed by the research team under the guidance of an associate professor with a Ph.D. in educational technology who had 1.5 years of visiting experience in English-speaking countries, and the Chinese wording was revised until consensus was reached. The process helped improve the semantic equivalence and contextual appropriateness of the translated and adapted questionnaire items.

To adapt the items to online learning environments, the researchers modified wording related to general learning, tasks, or activities so that the items specifically referred to online learning, while preserving the core meaning of the original scales. For instance, qualifiers such as “during online learning” or “in online learning activities” were added to make the items more context-specific. These adaptations clarified the online learning environments of the behaviors while preserving the original construct meanings.

Before the formal survey, a pilot test was conducted with 60 undergraduate medical students to examine whether the questionnaire items could be clearly understood by the target respondents and whether the adapted wording was appropriate for online learning environments. After the pilot test, the research team reviewed the pilot responses and feedback under the guidance of an associate professor with a Ph.D. in educational technology and 1.5 years of visiting experience in English-speaking countries. Minor revisions were then made to improve the clarity, readability, and contextual appropriateness of the questionnaire items before the questionnaire was formally distributed. The pilot test was used to refine the questionnaire, and formal psychometric validation was conducted using the formal survey data. The reliability and validity of the scales were further examined using the formal survey data. The pilot test data were not included in the final data analysis.

### 3.4. Data Analysis

SPSS 26 was used for statistical analysis, and AMOS 26 was used to construct the measurement model and structural equation model (SEM). Based on the recommendations regarding SEM sample size by J. Wang and Wang (2019), a sample size of 100–150 is generally considered a commonly cited minimum for SEM analysis. They also suggested that sample size can be considered in relation to the number of observed variables, and that a widely accepted rule of thumb is 10 cases or observations per indicator variable as a lower bound for an adequate sample size. The measurement model included 28 observed indicators, suggesting a minimum sample size of 280 based on the 10:1 ratio criterion. The final valid sample comprised 371 medical students. The sample size exceeded the recommended lower bound and was therefore considered acceptable for SEM analysis.

Harman’s single-factor test and the unmeasured latent method factor approach were used to assess common method bias (Tho, 2017). Confirmatory factor analysis was performed to examine the measurement model. The path analysis of the hypotheses proposed was performed through the structural equation model. According to the steps proposed by Baron and Kenny (1986), the mediation effect analysis was performed, and the bootstrap was performed with 5000 resampling. An indirect effect was considered statistically significant when the 95% confidence interval did not include zero.

## 4. Results

### 4.1. Convergent Validity, Composite Reliability and Discriminant Validity Test

According to the literature (Wu et al., 2021; Ying & Han, 2024), standardized factor loadings greater than 0.5 are acceptable. The composite reliability (CR) values greater than 0.6 indicate acceptable internal consistency. Furthermore, according to Fornell and Larcker (1981), when composite reliability is adequate, an average variance extracted (AVE) value below 0.50 but above 0.40 may still be considered acceptable, such values indicate marginal but acceptable convergent validity. The same criterion has also been adopted by He et al. (2025).

The test results of the standardized factor loadings, average variance extracted, and composite reliability of the scale were shown in Table 6. The standardized factor loadings of the constructs were between 0.536 and 0.859. The AVE values of all constructs were above 0.40, and the CR values were above 0.60. Although some AVE values were below the commonly recommended threshold of 0.50, the corresponding CR values and standardized factor loadings were acceptable. Therefore, the convergent validity of these constructs was considered marginal but acceptable.

Discriminant validity reflects the extent to which constructs are empirically distinct from one another (Saeed et al., 2019). The research employed the Heterotrait–Monotrait Ratio (HTMT) to assess discriminant validity, and the results are presented in Table 7. Based on the threshold recommended by Cheung et al. (2024), HTMT values below 0.85 indicate acceptable discriminant validity between constructs. As shown in Table 7, the HTMT values for all construct pairs were below 0.85, indicating that the measurement model had good discriminant validity.

### 4.2. Analysis Results of Measurement Model and Common Method Bias

**Figure 3:**
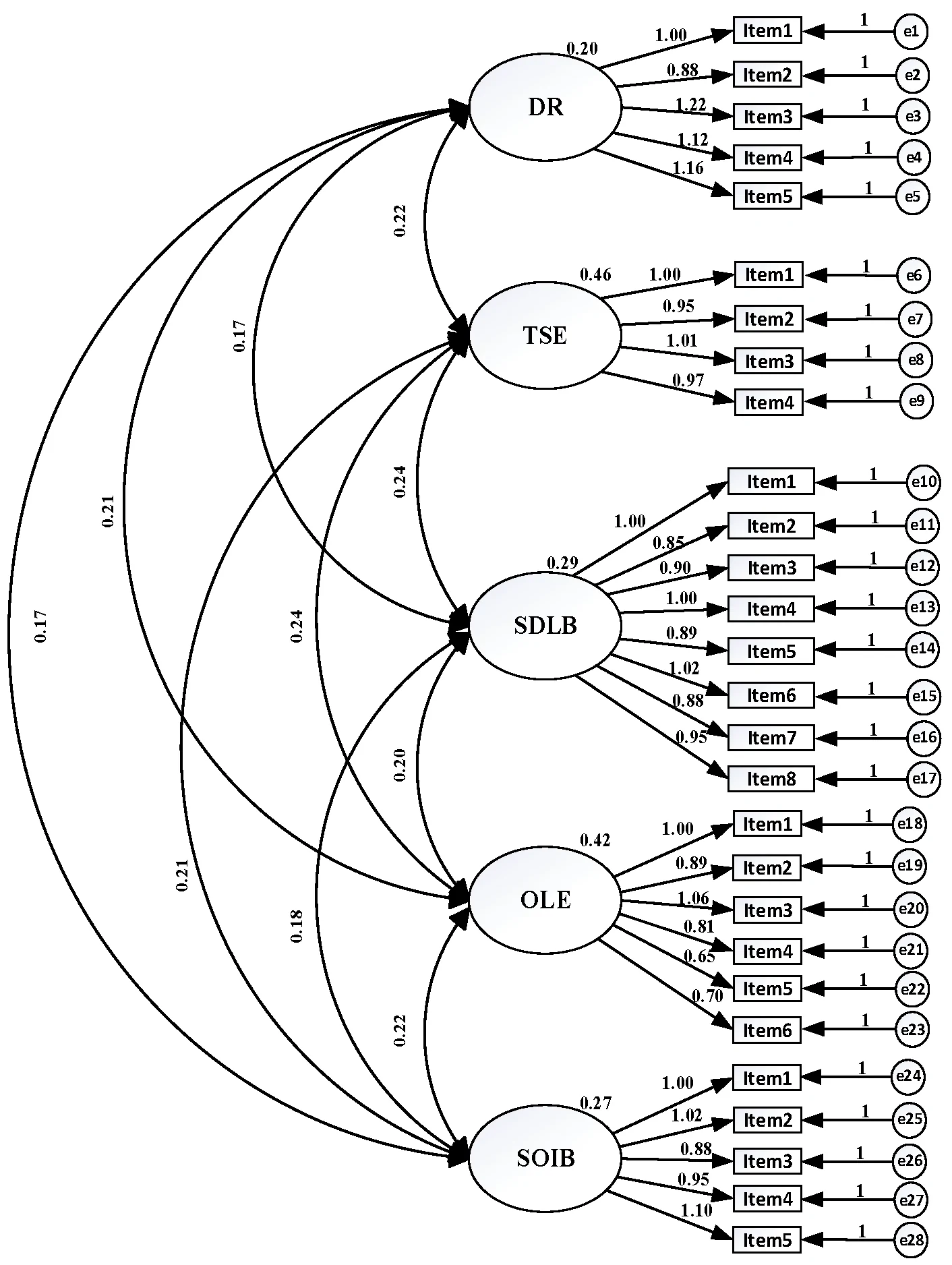
Measurement model in the research. Note. DR: Digital Resilience, TSE: Technological Self-efficacy, SDLB: Self-directed Learning Behaviors, OLE: Online Learning Engagement, SOIB: Self-reported Online Innovative Behavior.

The measurement model is represented by five latent constructs, as shown in Figure 3. The results of the evaluation of the measurement model are shown in Table 8. According to the reference range of the measurement model fit index (C. Li & Huang, 2024), the goodness of fit value of the measurement model is at an acceptable level.

Common method bias was first assessed using Harman’s single-factor test. The results showed that the first factor explained 37.885% of the total variance, which was below the commonly used threshold of 40%. The finding suggests that no single factor accounted for the majority of the variance. However, Harman’s single-factor test is only a preliminary diagnostic method and cannot conclusively rule out the possibility of common method bias, as common method bias is complex, widespread, and difficult to fully eliminate, particularly in studies relying on single-source self-reported data (Podsakoff et al., 2024). Therefore, the research further examined common method bias using the unmeasured latent method factor approach within the measurement model.

To further assess the potential influence of common method bias, the original measurement model was first specified with five substantive latent constructs, namely digital resilience, technological self-efficacy, self-directed learning behaviors, online learning engagement, and self-reported online innovative behavior. In the original measurement model, each observed item loaded only on its corresponding theoretical construct. Then, a common method factor model was specified by adding an unmeasured latent method factor to the original measurement model. In the model, each observed item was allowed to load on both its corresponding substantive construct and the unmeasured latent method factor. For model identification, the variance of the method factor was fixed to 1, and the unstandardized loadings from the method factor to all observed items were constrained to be equal. The method factor was specified as uncorrelated with the five substantive latent constructs.

The original measurement model and the common method factor model were compared using changes in model fit indices, including CFI, TLI, and RMSEA. According to the criteria recommended by Z. Huang et al. (2025) and Yang et al. (2025), if the addition of a common method factor leads to a substantial improvement in model fit, such as ΔCFI and ΔTLI greater than 0.10 or an absolute change in RMSEA greater than 0.05, it may indicate serious common method bias. As shown in Table 9, the changes in model fit indices were below the recommended thresholds (ΔCFI = 0.010, ΔTLI = 0.013, and ΔRMSEA = 0.006), suggesting that common method bias was unlikely to substantially affect the main findings of the research.

### 4.3. Descriptive Statistics, Correlation Analysis

The results of the correlation analysis are shown in Table 10. Self-directed learning behaviors are significantly positively correlated with technological self-efficacy (r = 0.577, *p* < 0.01), digital resilience (r = 0.579, *p* < 0.01), and online learning engagement (r = 0.559, *p* < 0.01). Technological self-efficacy is significantly positively correlated with digital resilience (r = 0.612, *p* < 0.01), online learning engagement (r = 0.546, *p* < 0.01), and self-reported online innovative behavior (r = 0.533, *p* < 0.01). Digital resilience is significantly positively correlated with online learning engagement (r = 0.657, *p* < 0.01) and self-reported online innovative behavior (r = 0.604, *p* < 0.01). Online learning engagement is significantly positively correlated with self-reported online innovative behavior (r = 0.611, *p* < 0.01).

### 4.4. Analysis Result of Structural Equation Modeling (SEM)

#### 4.4.1. Path Coefficient Test

**Figure 4:**
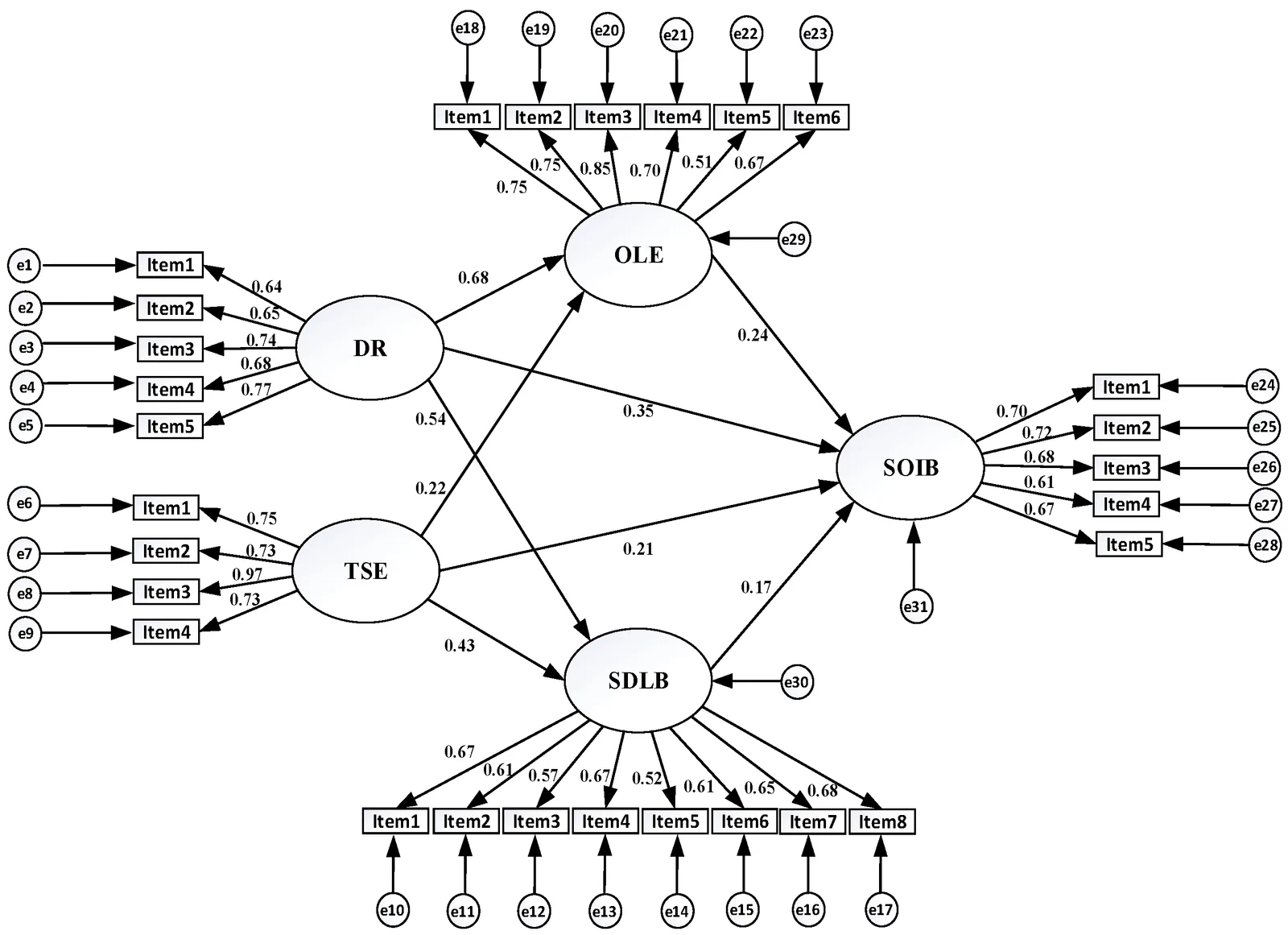
Structural model standardized path coefficients. Note. DR: Digital Resilience, TSE: Technological Self-efficacy, SDLB: Self-directed Learning Behaviors, OLE: Online Learning Engagement, SOIB: Self-reported Online Innovative Behavior.

According to the hypothesis contents, the constructed structural model is shown in Figure 4, and the corresponding model fit test results and path relationship hypothesis test results are shown in Table 11 and Table 12, respectively. According to the reference range of indicators used in the relevant literature, the corresponding indicators of the structural equation model fit index in Table 11 are all within the acceptable range.

The R^2^ values were examined to show the proportion of variance explained for each endogenous construct. As shown in Table 12, the model explained 47.8% of the variance in self-directed learning behaviors, 50.5% of the variance in online learning engagement, and 52.5% of the variance in self-reported online innovative behavior.

From the path analysis results in Figure 4 and Table 12, digital resilience was positively associated with self-directed learning behaviors. The unstandardized estimate was 0.491 (S.E. = 0.058, C.R. = 8.466, *p* < 0.001), and the standardized estimate was 0.540. Digital resilience was positively associated with online learning engagement. The unstandardized estimate was 0.791 (S.E. = 0.078, C.R. = 10.177, *p* < 0.001), and the standardized estimate was 0.676. Digital resilience was also positively associated with self-reported online innovative behavior. The unstandardized estimate was 0.318 (S.E. = 0.083, C.R. = 3.821, *p* < 0.001), and the standardized estimate was 0.348. These results provide empirical support for Hypothesis 1.

Technological self-efficacy was positively associated with self-directed learning behaviors. The unstandardized estimate was 0.327 (S.E. = 0.045, C.R. = 7.271, *p* < 0.001), and the standardized estimate was 0.432. Technological self-efficacy was positively associated with online learning engagement. The unstandardized estimate was 0.213 (S.E. = 0.042, C.R. = 5.097, *p* < 0.001), and the standardized estimate was 0.219. Technological self-efficacy was also positively associated with self-reported online innovative behavior. The unstandardized estimate was 0.157 (S.E. = 0.048, C.R. = 3.303, *p* < 0.001), and the standardized estimate was 0.206. These results provide empirical support for Hypothesis 2.

In addition, online learning engagement was positively associated with self-reported online innovative behavior. The unstandardized estimate was 0.190 (S.E. = 0.055, C.R. = 3.461, *p* < 0.001), and the standardized estimate was 0.243. Self-directed learning behaviors were also positively associated with self-reported online innovative behavior. The unstandardized estimate was 0.176 (S.E. = 0.076, C.R. = 2.313, *p* < 0.05), and the standardized estimate was 0.175. These results indicated that paths m1 and m2 were statistically significant in the hypothesized model.

#### 4.4.2. Test of Mediation Effect

The research examined whether digital resilience and technological self-efficacy are positively associated with self-reported online innovative behavior, and whether online learning engagement and self-directed learning behaviors mediate these associations. The path analysis results indicated that digital resilience and technological self-efficacy were positively associated with online learning engagement and self-directed learning behaviors. In addition, these constructs showed significant positive associations with self-reported online innovative behavior. The corresponding effects of digital resilience and technological self-efficacy in the hypothesized model are presented in Table 13 and Table 14, respectively.

The analysis results showed that digital resilience and technological self-efficacy had significant direct and indirect effects on medical students’ self-reported online innovative behavior. Within the hypothesized model, the total effect of digital resilience on medical students’ self-reported online innovative behavior was 0.554 (unstandardized estimate, *p* < 0.001, 95% CI = [0.430, 0.698]). The indirect effect of digital resilience accounted for 42.6% of the total effect of digital resilience, with an unstandardized estimate of 0.236 (*p* < 0.01, 95% CI = [0.115, 0.403]). The direct effect of digital resilience accounted for 57.4% of the total effect of digital resilience, with an unstandardized estimate of 0.318 (*p* < 0.01, 95% CI = [0.132, 0.511]). The total effect of technological self-efficacy on medical students’ self-reported online innovative behavior was 0.255 (unstandardized estimate, *p* < 0.001, 95% CI = [0.151, 0.385]). The indirect effect of technological self-efficacy accounted for 38.4% of the total effect of technological self-efficacy, with an unstandardized estimate of 0.098 (*p* < 0.01, 95% CI = [0.038, 0.191]). The direct effect of technological self-efficacy accounted for 61.6% of the total effect of technological self-efficacy, with an unstandardized estimate of 0.157 (*p* < 0.01, 95% CI = [0.051, 0.295]). These findings suggest that digital resilience and technological self-efficacy were positively associated with medical students’ self-reported online innovative behavior through both direct and indirect pathways.

Further analysis of the indirect effects revealed that digital resilience was indirectly associated with self-reported online innovative behavior through both online learning engagement and self-directed learning behaviors. The indirect effect through online learning engagement accounted for 27.1% of the total effect of digital resilience, with an unstandardized estimate of 0.150 (*p* < 0.01, 95% CI = [0.062, 0.278]). The indirect effect through self-directed learning behaviors accounted for 15.6% of the total effect of digital resilience, with an unstandardized estimate of 0.086 (*p* < 0.05, 95% CI = [0.005, 0.200]). Similarly, technological self-efficacy was indirectly associated with self-reported online innovative behavior through both online learning engagement and self-directed learning behaviors. The indirect effect through online learning engagement accounted for 15.9% of the total effect of technological self-efficacy, with an unstandardized estimate of 0.040 (*p* < 0.01, 95% CI = [0.014, 0.089]). The indirect effect through self-directed learning behaviors accounted for 22.5% of the total effect of technological self-efficacy, with an unstandardized estimate of 0.057 (*p* < 0.05, 95% CI = [0.006, 0.138]). These results suggest that online learning engagement and self-directed learning behaviors served as statistically significant mediating constructs in the associations among digital resilience, technological self-efficacy, and medical students’ self-reported online innovative behavior, thereby providing empirical support for Hypotheses 3, 4, 5, and 6.

## 5. Discussion

### 5.1. The Relationship Between Digital Resilience and Self-Reported Online Innovative Behavior

Students’ digital resilience in online learning environments was significantly and positively associated with students’ self-reported online innovative behavior. It is consistent with the relationship between student resilience and innovative behavior (Caniëls et al., 2022). Compared with traditional teaching environments, online learning environments provide students with greater flexibility and convenience in learning, but they are also accompanied by potential risks and challenges. For example, unfamiliarity with online learning platforms, harmful information, and privacy risks in online environments are potential factors associated with poorer online learning experiences (Lee & Hancock, 2023). Higher digital resilience is associated with students’ ability to cope with pressures in online learning environments and to use online resources to acquire knowledge, complete tasks, and achieve learning goals (Qi & Yang, 2024; Q. Pan et al., 2024). Because innovation often involves stress, uncertainty, and the risk of failure, digital resilience provides a useful perspective for understanding how students cope with difficulties and use online learning resources during learning activities related to innovation.

### 5.2. The Relationship Between Technological Self-Efficacy and Self-Reported Online Innovative Behavior

The results indicated that students’ technological self-efficacy in online learning environments was significantly and positively associated with their self-reported online innovative behavior. The finding is consistent with previous research showing a positive correlation between self-efficacy and students’ innovative behavior (Gupta & Singh, 2014). Students need a certain level of technical proficiency to effectively access learning resources and interact with teachers or other students (Rahman et al., 2023). With the development of digital technology, online learning platforms have become increasingly multifunctional (Ellis & Bliuc, 2019). Innovation in online learning environments requires students to use platform functions such as resource retrieval, course learning, and teacher–student communication, as well as other technology-enabled tools (L. Pan et al., 2024). Technological self-efficacy reflects students’ confidence in using online learning platforms and participating in learning activities supported by technology. Masry-Herzallah and Watted (2024) reported that technological self-efficacy was positively correlated with students’ innovative behavior. Based on previous studies and the hypothesized model, technological self-efficacy is considered an important factor associated with students’ adaptation to online learning environments, their effective use of digital tools, and their self-reported online innovative behavior.

### 5.3. The Indirect Associations Through Self-Directed Learning Behaviors

The path analysis results indicated that self-directed learning behaviors were significantly and positively associated with students’ self-reported online innovative behavior. In addition, statistically significant indirect associations were found between digital resilience and students’ self-reported online innovative behavior through self-directed learning behaviors, and between technological self-efficacy and students’ self-reported online innovative behavior through self-directed learning behaviors. The result suggests that self-directed learning behaviors are related to the associations of students’ adaptive capacity and confidence in digital environments with their self-reported online innovative behavior.

In online learning environments, the use of technology has become a routine part of students’ learning. Technological self-efficacy is a key construct associated with whether students believe they can use technology to learn and achieve expected learning outcomes (X. Pan, 2020). In a technological environment, technological self-efficacy is significantly associated with students’ self-directed learning behaviors (An et al., 2022). However, digital technology in education has a dual nature. While it can support learning, inappropriate use may reduce students’ learning effectiveness or even undermine their learning outcomes (Selwyn, 2016).

Digital resilience reflects students’ capacity to cope with the potential negative effects of digital technology and to maintain active learning regulation in digital environments (P. Jin et al., 2024). The positive associations of digital resilience and technological self-efficacy with students’ self-directed learning behaviors indicate that students’ adaptive capacity and confidence in technological environments are closely linked to their active regulation of learning. The finding can be interpreted from the perspective of self-directed learning, which emphasizes learners’ autonomous goal setting, strategy use, resource seeking, monitoring, and regulation of learning processes. Learners with self-directed learning behaviors have the determination to achieve their goals and can continuously learn to obtain the necessary knowledge, skills, and abilities (Bergamin et al., 2019). In online learning environments, effective self-directed learning behaviors are associated with students’ ability to acquire online learning resources, develop self-directed learning habits and attitudes, and achieve better online learning outcomes (Ji et al., 2025). Self-directed learning ability is also closely associated with students’ innovation-related outcomes, including innovation ability and creativity (Qian et al., 2023). Therefore, students’ self-directed learning behaviors and their use of online platforms deserve further attention in future studies on students’ self-reported online innovative behavior. However, these indirect associations should be interpreted as statistical relationships, and causal mechanisms should not be inferred.

### 5.4. The Indirect Associations Through Online Learning Engagement

The path analysis results obtained indicated a significant positive association between online learning engagement and medical students’ self-reported online innovative behavior. Furthermore, statistically significant indirect associations were found between digital resilience and students’ self-reported online innovative behavior through online learning engagement, and between technological self-efficacy and students’ self-reported online innovative behavior through online learning engagement. The result suggests that online learning engagement is related to the associations of students’ digital resilience and technological self-efficacy with their self-reported online innovative behavior.

In online learning environments, students’ online learning engagement plays a vital role in learning outcomes (Heflin et al., 2017). Students with higher online learning engagement tend to engage more actively in learning tasks and goal achievement (Vezne et al., 2023), which provides a favorable basis for understanding their self-reported online innovative behavior. Such engagement provides a useful perspective for understanding students’ reported tendency to identify problems, explore learning resources, propose new ideas, and translate innovative thinking into learning actions. Technological self-efficacy is positively associated with students’ online learning engagement, as it reflects their confidence in using digital tools to participate in learning activities (Derakhshan & Zhang, 2024). The path analysis results indicated that digital resilience and technological self-efficacy were significantly and positively associated with students’ self-reported online innovative behavior, and that statistically significant indirect associations were found through online learning engagement. Therefore, the findings provide possible directions for future research on students’ self-reported online innovative behavior in online learning environments. Future studies could further examine activity design and learning content related to students’ online learning engagement, as online learning engagement was found to be positively associated with students’ self-reported online innovative behavior. These indirect associations should also be interpreted as statistical relationships, and causal mechanisms should not be inferred.

### 5.5. Implications of the Findings for Medical Education

The findings have specific implications for medical education. Modern medical education requires students not only to acquire theoretical knowledge but also to develop clinical reasoning skills, problem-solving abilities, patient-safety awareness, professional competence, and the ability to apply digital technologies. As online case-based learning, virtual simulation, virtual patients, and digitally mediated clinical learning activities are increasingly incorporated into medical education, students need to manage technical difficulties, process complex clinical information, and complete demanding learning tasks. Digital resilience and technological self-efficacy are relevant to students’ ability to adapt to online learning environments, use digital platforms confidently, and remain engaged in clinically relevant learning activities.

The statistically significant indirect associations through self-directed learning behaviors and online learning engagement suggest that the relationships of digital resilience and technological self-efficacy with self-reported online innovative behavior are linked to processes related to active learning. Clinical reasoning and clinical problem-solving often require students to identify problems, search for evidence, integrate information from multiple sources, and make reasoned judgments under conditions of uncertainty. Similar learning processes are involved in online case discussions, virtual simulation exercises, patient-safety scenarios, and other technology-supported clinical learning tasks. In these contexts, self-reported online innovative behavior can be understood as students’ reported tendency to generate new ideas, seek alternative solutions, improve learning approaches, and share ideas during online medical learning activities. Although the research did not directly assess clinical reasoning, patient-safety performance, professional competence, actual clinical behavior, or objective innovative outcomes, the findings suggest that digital resilience, technological self-efficacy, self-directed learning behaviors, and online learning engagement are relevant learner characteristics and learning processes that deserve further attention in future studies on innovation-oriented online and blended medical education.

### 5.6. Institutional Characteristics and Generalizability of the Findings

Chinese medical education includes professional curricula, clinical skills training, and clinical practice. Compared with students in non-medical institutions, medical students are situated in a specialized educational context that emphasizes professional knowledge, clinical competence, and professional attributes (W. Wang, 2021). Because all participants were recruited from a single medical university in China, the institutional characteristics of medical education should be considered when interpreting the findings. These characteristics may influence students’ online learning experiences, their use of digital technologies, and the ways in which they perceive and report their online innovative behavior. Therefore, the associations and indirect associations observed should be interpreted within the specific institutional context of medical education. Caution is needed when generalizing the findings to students from non-medical institutions or other educational contexts, and further validation in different institutional settings is warranted.

## 6. Conclusions, Limitations and Implications

The research examined the associations between undergraduate medical students’ digital resilience, technological self-efficacy, and self-reported online innovative behavior in online learning environments, as well as the statistically significant indirect associations through self-directed learning behaviors and online learning engagement. The conclusions apply to the associations observed in a convenience sample of undergraduate medical students from one medical university in China. The outcome reflects students’ self-reported online innovative behavior, not objectively assessed innovative performance. The results showed that: (1) Digital resilience and technological self-efficacy were significantly and positively associated with students’ self-reported online innovative behavior. (2) Statistically significant indirect associations were found between digital resilience and self-reported online innovative behavior through self-directed learning behaviors, and between technological self-efficacy and self-reported online innovative behavior through self-directed learning behaviors. (3) Statistically significant indirect associations were found between digital resilience and self-reported online innovative behavior through online learning engagement, and between technological self-efficacy and self-reported online innovative behavior through online learning engagement.

Based on the findings, the following possible directions are proposed for future intervention studies related to medical students’ self-reported online innovative behavior in online learning environments. (1) Future studies could examine whether online courses and optimized platform functions are associated with students’ adaptation to online learning environments, confidence in using digital technologies, and self-reported online innovative behavior. (2) Future studies could examine whether the interactive features of online platforms are associated with students’ learning interests, enthusiasm for participation, online learning engagement, and self-reported online innovative behavior. (3) Future studies could also examine the role of self-directed learning tasks, such as problem-based learning and project-based learning, in students’ active exploration, problem solving, and self-reported online innovative behavior. (4) Future studies could further examine whether platforms for displaying students’ innovative learning outcomes are associated with students’ willingness to share ideas, reflect on learning outcomes, and self-reported online innovative behavior.

Several limitations should be noted. First, the cross-sectional design does not allow temporal ordering or causal relationships to be inferred. It also cannot capture dynamic changes or long-term trends in the constructs. In future longitudinal research, the correlation between individual psychological characteristics and their long-term change trends will be taken into account. Second, the participants were undergraduate students from a single medical university in China, which may limit the generalizability of the findings. The institutional characteristics, such as curriculum structure, online teaching platforms, assessment methods, and available resources supporting learning and innovation, could influence students’ online learning experiences and their self-reported online innovative behavior. In addition, first-year and second-year students accounted for 88.7% of the valid sample. Students in different academic years may differ in curriculum, clinical learning experience, familiarity with online learning, and opportunities to engage in innovative learning activities. Therefore, the findings may more strongly reflect the experiences of lower-year medical students, and caution should be exercised when generalizing the results to students from other institutions, regional contexts, or higher academic years. Third, the research used a questionnaire survey to collect data on students’ psychological states, learning behaviors, and self-reported online innovative behavior. Although self-reported questionnaires are useful for capturing students’ perceptions and learning experiences, they may be influenced by social desirability, recall bias, and subjective interpretation. More importantly, the research did not directly observe students’ actual innovative behaviors, innovative products, or innovative achievements in online learning environments. Therefore, the outcome represents students’ self-reported online innovative behavior, not objectively observed innovative behavior, innovative products, or innovative achievements.

In future research, the sample range could be further expanded to collect data from students in different regions, different disciplines, and different educational stages. In addition, in future study, mixed data collection methods could be further adopted, such as combination of interview method and questionnaire survey method, and embedding student behaviors, facial expressions, and attention collection functions into online learning platforms. Such approaches could provide more robust evidence for understanding students’ self-reported online innovative behavior in online learning environments.

## Figures and Tables

**Table 1:** Digital resilience scale adapted for online learning environments.

Dimensions of Digital Resilience in Kurniadi et al. (2022)	Adapted Items in Online Learning Environments
Personal competence, high standards, and tenacity.	I am able to adapt to the online learning environment.
Trust in one’s instinct, tolerance of negative affect	During online learning, I do not give up when I encounter difficult-to-understand problems.
Positive acceptance of change and secure relationship	I am able to persist in using the online platform for learning.
Control	I know how to learn more effectively using online platforms.
Spiritual influence	I am confident in my ability to master new knowledge through online learning.

**Table 2:** Technological self-efficacy scale adapted for online learning environments.

Dimensions of Technological Self-Efficacy in C.-H. Wang et al. (2013)	Adapted Items in Online Learning Environments
The use of online learning platforms	I am confident in using online learning platforms for learning.
The content of online learning platforms	I am confident in learning the content on online platforms.
The functional interaction of online learning platforms	I am confident in using the interactive functions of online learning platforms.
The discussion in online learning platforms	I am confident in participating in online learning discussions.

**Table 3:** Online learning engagement scale adapted for online learning environments.

Dimensions of Online Learning Engagement in J. C.-Y. Sun and Rueda (2012)	Adapted Items in Online Learning Environments
Behavioral engagement	I follow the rules of the online course.
	When learning online, I only “pretend” to study.
	I am able to stay focused while learning online.
Emotional engagement	I find online learning interesting.
	I am interested in the content of the online course.
	I enjoy the online course.
Cognitive engagement	I engage in online learning on my own initiative.
	I read additional learning resources to better understand the course content.
	When I encounter difficulties during online learning, I find ways to figure them out.

Note. The item “When learning online, I only ‘pretend’ to study” was reverse coded.

**Table 4:** Self-directed learning behaviors scale adapted for online learning environments.

Dimensions of Self-Directed Learning Behaviors in Lin et al. (2017)	Adapted Items in Online Learning Environments
Goal-setting	I consistently hold myself to high standards in online courses.
	I set learning goals to better plan my study time for online courses.
Task strategies	I take notes as carefully as possible in online courses, as note-taking is more important for online learning than in a traditional classroom.
	In addition to solving the questions assigned by the teacher, I also solve additional problems to better grasp the content of the online courses.
Help-seeking	I find someone who is familiar with the course content and ask them for help when needed.
	I share my questions with classmates online so that we can understand what we are learning and how to solve our problems.
Self-evaluation	While learning online, I summarize regularly to check my understanding of the content.
	When studying online courses, I ask myself many questions about the learning content.

**Table 5:** Self-reported online innovative behavior scale adapted for online learning environments.

Items of Innovative Behavior Scale in Iddris et al. (2026)	Adapted Items in Online Learning Environments
I develop ideas in new ways to solve difficult problems.	When learning online, I use new ways of thinking to solve difficult problems.
I try to find new ways to be used to perform my work.	When learning online, I try to find new methods to complete learning tasks.
I consider creative solutions to work-related problems.	When learning online, I seek creative solutions when I encounter problems.
I will introduce my new ideas to people around me to gain support and recognition.	When learning online, I share my new ideas with others to gain support and recognition.
I often come up with some innovative ideas.	When learning online, I frequently come up with innovative ideas.

**Table 6:** Results of convergent validity and composite reliability tests.

Latent Constructs	Standardized Factor Loadings of Observed Constructs	AVE	CR
SDLB	Item1 ← 0.728	0.447	0.866
	Item2 ← 0.657		
	Item3 ← 0.612		
	Item4 ← 0.694		
	Item5 ← 0.604		
	Item6 ← 0.667		
	Item7 ← 0.684		
	Item8 ← 0.692		
TSE	Item1 ← 0.844	0.674	0.892
	Item2 ← 0.831		
	Item3 ← 0.829		
	Item4 ← 0.777		
DR	Item1 ← 0.672	0.487	0.825
	Item2 ← 0.618		
	Item3 ← 0.738		
	Item4 ← 0.688		
	Item5 ← 0.763		
OLE	Item1 ← 0.766	0.537	0.872
	Item2 ← 0.786		
	Item3 ← 0.859		
	Item4 ← 0.722		
	Item5 ← 0.536		
	Item6 ← 0.684		
SOIB	Item1 ← 0.731	0.504	0.835
	Item2 ← 0.757		
	Item3 ← 0.718		
	Item4 ← 0.646		
	Item5 ← 0.694		

Note. DR: Digital Resilience, TSE: Technological Self-efficacy, SDLB: Self-directed Learning Behaviors, OLE: Online Learning Engagement, SOIB: Self-reported Online Innovative Behavior.

**Table 7:** Results of Heterotrait–Monotrait Ratio.

	SDLB	TSE	DR	OLE	SOIB
SDLB	-				
TSE	0.668	-			
DR	0.695	0.726	-		
OLE	0.676	0.648	0.814	-	
SOIB	0.661	0.626	0.739	0.752	-

Note. DR: Digital Resilience, TSE: Technological Self-efficacy, SDLB: Self-directed Learning Behaviors, OLE: Online Learning Engagement, SOIB: Self-reported Online Innovative Behavior.

**Table 8:** Goodness of fit indices of measurement model.

	x^2^/df	NFI	CFI	IFI	TLI	RMSEA	PNFI	PGFI
Recommended values (C. Li & Huang, 2024)	<3.0	>0.9	>0.9	>0.9	>0.9	<0.10	>0.5	>0.6
Model values	1.624	0.909	0.963	0.963	0.952	0.041	0.707	0.749

**Table 9:** Comparison of model fit between the original measurement model and the common method factor model.

	x^2^/df	CFI	TLI	RMSEA	ΔCFI	ΔTLI	ΔRMSEA
Measurement model values	1.624	0.963	0.952	0.041	0.01	0.013	0.006
Values of measurement model with common method factor	1.448	0.973	0.965	0.035			

**Table 10:** The Results of Descriptive Statistics and Correlation Analysis.

	M	SD	SDLB	TSE	DR	OLE	SOIB
SDLB	3.7692	0.53581	1				
TSE	3.6914	0.69074	0.577 **	1			
DR	3.883	0.52071	0.579 **	0.612 **	1		
OLE	3.668	0.54847	0.559 **	0.546 **	0.657 **	1	
SOIB	3.7261	0.55277	0.558 **	0.533 **	0.604 **	0.611 **	1

Note. *** *p* < 0.001; ** *p* < 0.01; * *p* < 0.05. DR: Digital Resilience, TSE: Technological Self-efficacy, SDLB: Self-directed Learning Behaviors, OLE: Online Learning Engagement, SOIB: Self-reported Online Innovative Behavior.

**Table 11:** Goodness of fit indices of Structural model.

Goodness of Fit	Fit Criteria	Values of the Model
x^2^/df (Sümer, 2000)	0 ≤ x^2^/df ≤ 5	2.163
RMSEA (MacCallum et al., 1996)	0 ≤ RMSEA ≤0.10	0.056
GFI (Schermelleh-Engel et al., 2003)	0.90 ≤ GFI ≤ 0.95	0.902
CFI (Hu & Bentler, 1999)	0.90 ≤ CFI ≤ 0.95	0.931
IFI (Bentler & Bonett, 1980)	0.90 ≤ IFI ≤ 0.95	0.932
AGFI (Schermelleh-Engel et al., 2003)	0.85 ≤ AGFI ≤ 0.90	0.863

**Table 12:** Results of structural model path relationship test.

Path	R^2^	Standardized Coefficients Estimate	Unstandardized Coefficients Estimate	S.E.	C.R.	*p* Value	Label
SDLB ← DR	0.478	0.540	0.491	0.058	8.466	***	a2
SDLB ← TSE		0.432	0.327	0.045	7.271	***	b2
OLE ← DR	0.505	0.676	0.791	0.078	10.177	***	a1
OLE ← TSE		0.219	0.213	0.042	5.097	***	b1
SOIB ← TSE	0.525	0.206	0.157	0.048	3.303	***	b3
SOIB ← DR		0.348	0.318	0.083	3.821	***	a3
SOIB ← OLE		0.243	0.190	0.055	3.461	***	m2
SOIB ← SDLB		0.175	0.176	0.076	2.313	0.021 *	m1

Note. *** *p* < 0.001; ** *p* < 0.01; * *p* < 0.05. DR: Digital Resilience, TSE: Technological Self-efficacy, SDLB: Self-directed Learning Behaviors, OLE: Online Learning Engagement, SOIB: Self-reported Online Innovative Behavior, R^2^: estimates of squared multiple correlations, S.E.: standard error; C.R.: critical ratio.

**Table 13:** Results for the total, direct, and indirect effects of digital resilience.

Parameter	Boot SE	Unstandardized Coefficients Estimate	Lower (95%)	Upper (95%)	*p* Value	Relative Effect Size Based on DR Total Effect(%)
Total effects of DR	0.069	0.554	0.430	0.698	***	-
Indirect effects of DR	0.073	0.236	0.115	0.403	0.001 **	42.6
Direct effects of DR	0.097	0.318	0.132	0.511	0.001 **	57.4
Indirect for path of DR→OLE→SOIB	0.054	0.150	0.062	0.278	0.001 **	27.1
Indirect for path of DR→SDLB→SOIB	0.050	0.086	0.005	0.200	0.037 *	15.6

Note. *** *p* < 0.001; ** *p* < 0.01; * *p* < 0.05. DR: Digital Resilience, SDLB: Self-directed Learning Behaviors, OLE: Online Learning Engagement, SOIB: Self-reported Online Innovative Behavior.

**Table 14:** Results for the total, direct, and indirect effects of technological self-efficacy.

Parameter	Boot SE	Unstandardized Coefficients Estimate	Lower (95%)	Upper (95%)	*p* Value	Relative Effect Size Based on TSE Total Effect(%)
Total effects of TSE	0.060	0.255	0.151	0.385	***	-
Indirect effects of TSE	0.038	0.098	0.038	0.191	0.002 **	38.4
Direct effects of TSE	0.060	0.157	0.051	0.295	0.003 **	61.6
Indirect for path of TSE→OLE→SOIB	0.018	0.040	0.014	0.089	0.001 **	15.9
Indirect for path of TSE→SDLB→SOIB	0.033	0.057	0.006	0.138	0.031 *	22.5

Note. *** *p* < 0.001; ** *p* < 0.01; * *p* < 0.05. TSE: Technological Self-efficacy, SDLB: Self-directed Learning Behaviors, OLE: Online Learning Engagement, SOIB: Self-reported Online Innovative Behavior.

## Data Availability

The datasets used and/or analysed during the current study are available from the corresponding author on reasonable request.
